# Simultaneous EEG‐fMRI Reveals a Visual Working Memory Encoding Network Related to Theta Oscillatory Activity in Healthy Subjects

**DOI:** 10.1002/hbm.70216

**Published:** 2025-04-21

**Authors:** Gregor Leicht, Jonas Rauh, Marius Mußmann, Sebastian Vauth, Saskia Steinmann, Moritz Haaf, Corinna Haenschel, Christoph Mulert

**Affiliations:** ^1^ Department of Psychiatry and Psychotherapy, Psychiatry Neuroimaging Branch (PNB) University Medical Center Hamburg‐Eppendorf Hamburg Germany; ^2^ Department of Psychology City University of London London UK; ^3^ Center of Psychiatry Justus‐Liebig University Giessen Germany

**Keywords:** delayed match to sample task, DLPFC, encoding, simultaneous EEG‐fMRI, single‐trial coupling, theta oscillations, working memory

## Abstract

Working memory (WM) is crucially involved in many aspects of higher cognitive functions and goal‐directed behavior. The encoding of sensory information necessitates the conversion of sensory stimuli into maintainable constructs. Oscillatory activity in the theta frequency range (4–8 Hz) of the human electroencephalogram (EEG) has been related to this. However, so far, no study has investigated the neurophysiological mechanisms and the brain network structure underlying the WM encoding process simultaneously. Thus, this study aimed to test whether theta oscillatory activity would be specifically related to the activity within a WM encoding brain network in healthy subjects by means of simultaneous recordings of EEG and functional magnetic resonance imaging (fMRI). Simultaneous recordings of EEG and fMRI were conducted in 32 healthy subjects during the performance of a visual working memory delayed matched to sample task. The fMRI analysis was informed by single‐trial theta oscillatory responses to encoding stimuli. This analysis revealed a working memory encoding network mediated by theta oscillatory activity. The network included regions within the dorsolateral prefrontal cortex and parietal areas. Our results give reason to assume that the formation of a working memory network might take place during the encoding of information utilizing theta synchrony as a binding mechanism.

## Graphical abstract

By means of simultaneous recordings of EEG and fMRI, we identified a working memory encoding network mediated by theta oscillatory activity, including the dorsolateral prefrontal cortex and parietal areas. Our results suggest the formation of such a network during the encoding phase, possibly utilizing theta synchrony as a binding mechanism.

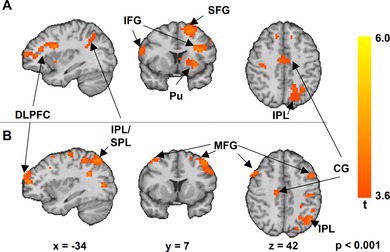

## 1 Introduction

Working memory (WM) is crucially involved in many aspects of higher cognitive functions and goal‐directed behavior. It comprises a set of operations that allow the simultaneous maintenance and manipulation of behaviorally relevant information over short periods of time. The most widely used conceptualization of working memory discriminates three distinct WM processes: encoding (of new information), maintenance (processes of storage and manipulation of information over time) and retrieval (recall or recognition of stored information) (Baddeley 2012). Recent evidence suggests distinct central processing mechanisms for these different working memory processes. Encoding of sensory information is assumed to represent the conversion of sensory stimuli into a maintainable construct when the stimulus is no longer accessible (Palva et al. 2011). Recently, impaired working memory capacities in neuropsychiatric disorders such as schizophrenia have been linked to abnormal WM encoding mechanisms in behavioral, electroencephalography (EEG), magnetoencephalography (MEG) and functional magnetic resonance imaging (fMRI) studies (Bittner et al. 2015; Cairo et al. 2006; Haenschel et al. 2007; Johnson et al. 2006; Kang et al. 2018; Lee and Park 2005; Lencz et al. 2003; Stablein et al. 2019).

The brain networks involved in WM processes have been extensively described. A meta‐analysis of 189 functional resonance imaging (fMRI) studies revealed a WM network including inferior frontal gyrus (IFG), middle frontal gyrus (MFG), anterior insula, precentral gyrus, (pre‐)SMA, dorsal premotor cortex, inferior parietal lobule (IPL) and superior parietal lobule (SPL) and subcortical areas (thalamus and basal ganglia) (Rottschy et al. 2012). Besides visual sensory brain areas, the encoding of visual information into WM has been attributed to frontal and parietal brain regions (Majerus et al. 2007; Munk et al. 2002) involving the bilateral intraparietal sulcus, the frontal eye fields, the SMA, the premotor cortex, and the middle and inferior frontal gyri (Linden et al. 2003).

Oscillatory components of brain activity have been suggested to be generated by network activity of synchronized cell assemblies (Buzsaki and Draguhn 2004) and are regarded as signatures of functional coupling between brain regions during distributed cognitive processes (Engel et al. 2001). Oscillatory activity in the theta frequency range (4–8 Hz) is typically considered as a marker of attention or memory processes during cognitively demanding tasks (Buzsaki 2005) and has frequently been related to working memory processes (Maurer et al. 2015; Roux and Uhlhaas 2014). Successful WM encoding involves task‐related increases of theta band activity (Klimesch 1999; Klimesch et al. 1996) and characteristics of evoked theta responses to visual objects during their encoding in WM have predicted the successful memorization of these objects (Haenschel et al. 2009). Theta has been suggested to be involved in the long‐range communication between brain regions during successful WM encoding. However, very little effort has been taken to relate the large body of fMRI WM literature to the localization of the generators of this specific neuronal oscillatory mode. Only one study reported EEG source localization results with respect to theta activity during WM encoding revealing cortical sources of increased theta activity during the encoding of visual–spatial information within the IFG (Jaiswal et al. 2010). This might be due to the limitations of EEG‐based approaches, resulting from the lack of a unique solution to the inverse problem of cortical source localizations based on scalp‐recorded activity. In fact, the characterization of both the network structure and the neurophysiological mechanisms involved is highly desirable, especially in view of recent attempts to improve WM deficits in psychiatric diseases using theta frequency transcranial alternating current stimulation (tACS) (Sreeraj et al. 2017). However, tACS studies show inconsistent findings, which might be due to rough estimates of stimulation sites (Hoy et al. 2016). The accurate localization of stimulation target regions necessitates the identification of the regions included in a WM theta oscillatory network. Simultaneous EEG‐fMRI provides the methodological basis for this goal using both the superiority of EEG in assessing the temporal characteristics of neural oscillations and the excellent spatial resolution of fMRI (Mulert et al. 2010; Mulert et al. 2008).

While different studies have used simultaneous recordings of EEG and fMRI in order to investigate WM retention (Baenninger et al. 2016; Michels et al. 2010; Mizuhara et al. 2015; Scheeringa et al. 2009) or the recollection of memories (Herweg et al. 2016), so far, only two EEG‐fMRI studies have reported a finding on theta oscillatory activity related to a WM encoding network: Zhao et al. showed a WM load dependent modulation of theta activity with a corresponding activation of frontal and parietal lobes related to information encoding during a n‐back task (Zhao et al. 2017). Forsyth et al. found a positive correlation of frontal midline theta activity with activity in the right superior frontal gyrus and an association of a load dependent theta power change with a decrease of the BOLD activity in the left inferior frontal gyrus and orbital cortex (Forsyth et al. 2021). However, both studies were not able to clearly separate the mechanisms of WM encoding, maintenance, and retrieval.

Prompted by the above, our study focuses on the description of a theta oscillatory working memory encoding network. We hypothesized that oscillatory theta activity evoked by visual stimuli during encoding into WM would be involved in a WM encoding brain network in healthy subjects. Furthermore, we hypothesized that the activity of this theta‐specific brain network would predict WM performance. In order to test these hypotheses, we investigated 32 healthy subjects using simultaneous recordings of EEG and fMRI during the performance of a visual working memory task, which was able to separate processes of encoding from WM maintenance and retrieval.

## 2 Materials and Methods

### 2.1 Ethics Approval Statement

The present study was part of a larger project investigating resting‐state and task‐related brain connectivity in schizophrenia by means of EEG, MEG, and simultaneous EEG‐fMRI, within the context of the Collaborative Research Centre 936 (“multi‐site communication in the brain”, www.sfb936.net). The study was approved by the Ethics Committee of the Medical Association Hamburg and carried out in accordance with the Helsinki Declaration of 1975, as revised in 2013. Written informed consent was obtained from all participants after the aim of the study and the nature of the procedures had been fully explained.

### 2.2 Participants

The study sample consisted of 32 healthy adults aged between 18 and 35 years (mean age: 23.0, SD: 4.3), including 18 female and 14 male participants. Exclusion criteria for all participants were any previous psychiatric disorder or treatment, a family history of psychotic disorders, current substance abuse, or dependence, and the presence of major somatic or neurological disorders. One male participant was excluded from the analyses due to bad MRI data quality.

### 2.3 Paradigm

A visual working memory delayed matched to sample reaction task adapted from Haenschel et al. (2007) and Linden et al. (2003) was used containing the presentation of 36 non‐natural visual objects (blurred outlines of random tetris shapes) in three conditions with varying working memory load. The advantage of these stimuli is their novelty and the absence of the possibility of verbalization.

**FIGURE 1:**
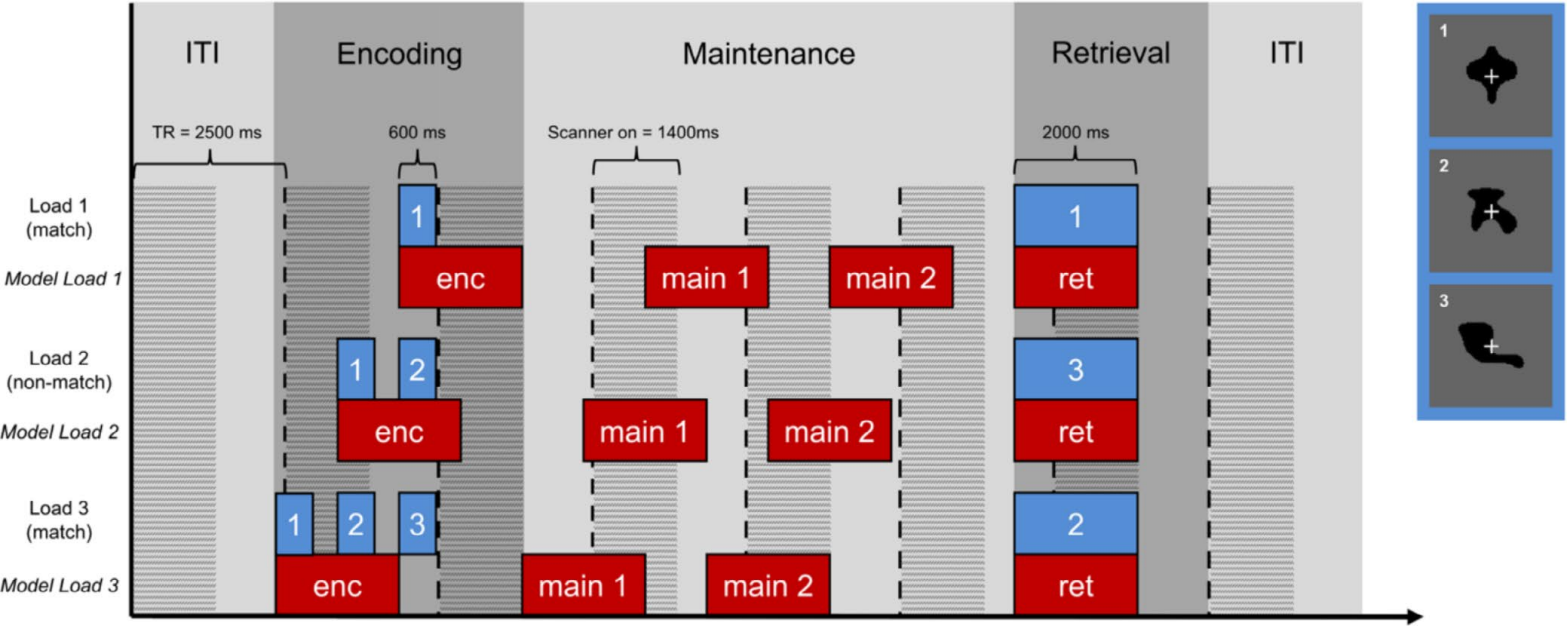
Delayed Match to Sample (DMTS) task and fMRI model. The WM periods (encoding (enc), maintenance (main), retrieval (ret)) are shown in different shades of grey. The individual stimuli are shown in blue, which correspond to exemplary stimuli on the right. The blocks of 2 s used in the fMRI model are shown in red. The periods of time with the scanner gradient switched on are shaded. The dashed lines mark the beginning of a new repetition time (TR). ITI, intertrial interval.

One trial consisted of an encoding, a maintenance and a retrieval phase (Figure 1). During the encoding phase one (WM load condition 1), two (condition 2) or three (condition 3) different visual objects were shown for 600 ms each with an interstimulus interval of 400 ms, resulting in a duration of the encoding phase of 1 to 3 s depending on the different conditions. Following the encoding phase, a fix‐cross was shown for 10 s. The participants were instructed to maintain the displayed items during this maintenance phase. In the following retrieval phase, a probe stimulus was shown for 2000 ms, and the participant was asked to indicate as fast and accurately as possible whether this probe stimulus had been shown during the encoding phase via button press with the left (not shown during encoding phase, mismatch) or the right (shown during encoding phase, match) index finger. The intertrial interval was 5000 ms. The whole experiment consisted of 120 trials (40 per condition) presented in two runs. The trials were presented in a pseudo‐randomized order.

### 2.4 Behavioral Analysis

In accordance with previous reports (Haenschel et al. 2009; Linden et al. 2003) the number of successfully encoded items was calculated using Pashler's formula (Pashler 1988): *s* = *n* × (*h*−*g*)/(1−*g*), where *s* is the number of stored items, *n* is the number of items in the display (WM load: 1 in condition 1, 2 in condition 2, etc.), *h* is the hit rate (correctly identified matches), and *g* is the rate of false alarms (non‐matches incorrectly identified as matches). Error rates were defined as the number of incorrect responses (sum of not identified matches and non‐matches incorrectly identified as matches) divided by the total number of trials.

### 2.5 fMRI

#### 2.5.1 fMRI Acquisition

Imaging was performed on a 3 T MRI scanner (Siemens Magnetom Trio) equipped with a 12‐channel head coil using a standard gradient echo‐planar imaging T2*‐sensitive sequence for functional BOLD imaging. 24 slices covering the whole brain (*TR* = 2.5 s; *TA* = 1.4 s; *TE* = 30 ms; *FOV* = 216/216 mm; matrix = 72 × 72; interleaved slice acquisition; slice thickness = 4 mm; interslice gap = 1 mm; resulting pixel size = 3 × 3 mm) were acquired in two sessions (482 volumes each) in the same position as a three‐dimensional MPRAGE data set (T1‐weighted). We used a “sparse sampling design” (Hall et al. 1999) including MR‐acquisition‐free periods of 1.1 s in every TR in order to gain EEG data free of MR gradient artifacts.

#### 2.5.2 fMRI Preprocessing

The processing of fMRI data was conducted using the BrainVoyager QX software package (version 2.8, Rainer Goebel, Maastricht, Netherlands). Slice time correction using an acquired slice time table (spline interpolation), motion correction (trilinear/sinc interpolation) and a temporal high‐pass GLM‐Fourier filtering including linear trend removal were conducted before alignment of the functional data with the three‐dimensional anatomical volumes, transformation into the Talairach space, interpolation to a resolution of 3 × 3 × 3 mm^3^ and Gaussian 3D spatial smoothing (8 mm kernel size, full‐width half‐maximum).

#### 2.5.3 fMRI Model and Analysis

For further statistical analysis, regression coefficients were estimated based on a general linear model (GLM). A multiple regression analysis was performed on the three‐dimensional functional volume time courses (one for each subject). Following a previous fMRI study using the same paradigm (Bittner et al. 2015), a corresponding design matrix including 12 regressors (four regressors for each of the three conditions) and a baseline period was modeled for each subject and convolved with a double‐gamma hemodynamic response function. Each of the four regressors was defined as a block of 2 s modeling (1) the encoding period starting at stimulus onset of the first encoding stimulus, (2) the early maintenance period starting 4 s after the onset of the encoding block, (3) the late maintenance period starting 7 s after the onset of the encoding block, and (4) the retrieval period starting at stimulus onset of the retrieval stimulus. Moreover, six translation and rotation vectors derived from the motion correction were added as confounds.

Within the general linear model framework, first‐level statistical maps containing estimated beta values were calculated for each subject. The resulting maps were entered into a second‐level group analysis treating inter‐subject variability as a random effect. In order to show overall activity related to the encoding period, a contrast combining the beta estimates of all three encoding regressors was calculated. Furthermore, in order to investigate working memory load dependent BOLD signal changes, a single‐factor repeated measures ANOVA including the estimates of all three encoding regressors was calculated. The resulting 3D statistical maps are presented at a significance level of *p* < 0.0001 (Bonferroni corrected for multiple comparisons). The mean beta estimates of each cluster of significant voxels from the ANOVA analysis were extracted and compared between each condition using *t*‐tests. Labeling of anatomical regions was based on the Talairach Daemon (http://www.talairach.org/daemon.html, Version 2.4.3).

### 2.6 EEG

#### 2.6.1 EEG Acquisition

EEG recordings took place within the MRI scanner during the acquisition of fMRI data. Subjects were lying on their back and were asked to keep the eyes open and look at a fixation cross projected to a mirror mounted on the head coil. We used two EEG amplifiers specifically developed for operation in the MRI scanner environment and particularly avoiding saturation by magnetic activity (BrainAmp MRplus; Brain Products, Munich, Germany). The EEG recording was performed in alternating current (AC) mode with 62 active EEG electrodes (sintered silver and silver‐chlorid) mounted on an elastic cap (BrainCapMR 64, Brain Products, Munich, Germany) using the BrainVision Recorder software (Version 1.20, Brain Products, Munich, Germany). Electrodes were arranged according to a modified 10/10 system (reference electrode: FCz; ground: AFz). The electrode skin impedance was kept below 10 kΩ. Data were collected with a sampling rate of 5000 Hz and an analogous band‐pass filter (0.1–250 Hz). The amplitude resolution was set to 0.5 μV per bit.

#### 2.6.2 EEG Preprocessing

The EEG data analysis was carried out using Brain Vision Analyzer (BVA) Version 2.1 (Brain Products, Munich, Germany). The MR‐Artifact was corrected by subtracting a sliding average template of 21 succeeding intervals using baseline correction (based on Allen et al. 2000). Subsequently, the data was filtered using a band‐pass (0.1–30 Hz, Butterworth Zero Phase Filter using a slope 12 dB/oct for high‐pass and 48 dB/oct for low‐pass filter). To reduce cardioballistic artifacts, R‐peaks measured at a dedicated ECG electrode on the subject's back were automatically detected using the function implemented in BVA. The proposed markers were visually inspected and in rare cases manually adjusted. Afterwards, BCG templates were calculated using the information on time points of R peaks and removed from the EEG signal using the algorithm implemented in the BVA (Allen et al. 1998). After resampling the data to a sampling rate of 500 Hz a cardioballistic artifact template formed out of 21 succeeding intervals following heartbeats was subtracted from every EEG channel at the position of heartbeat markers. In order to prepare data for an additional artifact rejection using Independent Component Analysis (ICA) an automatic raw data inspection (elimination of EEG intervals exceeding the following limits: max. voltage step: 30 μV/ms; max. allowed amplitude difference within interval length of 200 ms: 200 μV) was conducted. By means of ICA components containing residual gradient, blink or movement artifacts were identified and subsequently removed. After re‐referencing to a common average FCz electrode position data were interpolated using spherical splines. Previous studies Haenschel et al. (2007) and Koychev et al. (2011) have shown that electrophysiological responses to the encoding stimuli of our working memory task occur mainly in two clusters on the scalp, a fronto‐central (anterior) and a parieto‐occipital (posterior) cluster, which is in line with our findings. Therefore, in line with a study using the same paradigm (Haenschel et al. 2007), electrodes were then pooled into an anterior (C1, C2, Cz, F1, F2, FC1, FC2, FCz, and Fz) and a posterior (O1, O2, Oz, P3, P4, PO3, PO4, POz, and Pz) region‐of‐interest (ROI), each comprising 9 electrodes.

#### 2.6.3 EEG Analysis

For further analysis of the encoding phase data was segmented into epochs of 5.5 s starting 2.5 s before the onset of the first encoding stimulus. Trials with an incorrect or no answer within a timeframe of 6 s following the onset of the retrieval stimulus were discarded. A baseline correction was applied using a baseline of 2500 ms before the onset of the first encoding stimulus of a trial. For the purpose of artifact rejection, all epochs exceeding a voltage of ±80 μV were discarded. For every subject, an average of the residual epochs was calculated.

In order to yield time‐frequency information, a continuous wavelet transformation using a complex Morlet wavelet (real values [μV^2^], 25 frequency layers distributed on a logarithmic scale, Morlet parameter *c* = 5, Gabor Normalization, normed output relative to baseline) was used as done previously (Leicht et al. 2010). In order to parametrize theta band activity wavelet layers centered at 5 Hz were calculated. For replication of previous results (Haenschel et al. 2009) peak evoked theta activity (5 Hz) during the encoding phase was defined as the highest theta power value within the timeframe between 30 and 550 ms after the onset of the last encoding stimulus. In order to check for WM load effects with respect to theta activity across all presented encoding stimuli (as used in the EEG‐informed fMRI analysis), mean theta peak values for two encoding stimuli in condition 2 and for three encoding stimuli in condition 3 were calculated.

### 2.7 EEG‐Informed fMRI Analysis

In order to obtain single trial theta power values for the EEG‐informed analysis, the aforementioned wavelet transformation with frequency layers centered at 5 Hz was conducted on the single trial level without preceding artifact rejection and averaging of the single trial EEG segments. For every single trial, for every encoding stimulus (1, 2, or 3 per trial, depending on the condition) and for each of the two ROIs theta power peak values were detected as the highest values within the aforementioned peak detection timeframe (30 to 550 ms after the onset of the respective encoding stimulus). For conditions 2 and 3, mean theta peak values per trial were calculated across all encoding stimuli presented in this trial (out of two values in condition 2 and out of three values in condition 3). This resulted in two different time series comprising information on oscillatory activity evoked during visual WM encoding: (1) in the theta frequency range within the anterior pool of electrodes and (2) in the theta frequency range within the posterior pool of electrodes. Values that exceeded more than 2 standard deviations of the mean of the respective time series were considered artifacts and replaced by the mean of single trial values of the corresponding time series. The resulting time series were used to calculate regressors for the EEG‐informed fMRI GLM analysis.

For that purpose, the time series of encoding periods (one for each of the three conditions, encoding phases represented by a constant amplitude of 1), which were included in the encoding period regressor (stimulus functions *X*_*E1*_, *X*_*E2*_, and *X*_*E3*_) as defined in the section *fMRI parameter and analysis*, were replaced by the time series of theta power peaks evoked by encoding stimuli. This resulted in two different functions per condition (e.g., for condition 1: *X*_*E1‐Theta‐Anterior*_ and *X*_*E1‐Theta‐Posterior*_) representing theta power variations over trials, while *X*_*E1*_, *X*_*E2*_, and *X*_*E3*_ represented encoding‐related BOLD activation not related to the theta power variation. In order to identify fMRI results specifically related to encoding processes involving theta oscillatory activity rather than a summarizing picture of working memory encoding, we used Schmidt‐Gram orthogonalization as suggested previously (Eichele et al. 2005) and applied by our and other groups in several studies (Andreou et al. 2017; Goldman et al. 2009; Leicht et al. 2016; Li et al. 2019; Mulert et al. 2008; Schauer et al. 2021). In the present study, the functions representing theta power variations over trials were orthogonalized with respect to *X*_*E1*_, *X*_*E2*_, or *X*_*E3*_, respectively, removing that part of these functions that was correlated to *X*_*E1*_, *X*_*E2*_, or *X*_*E3*_ (for a detailed description please see Mulert et al. (2010), orthogonalization was done using a function of the NeuroElf software v1.0 (www.neuroelf.net) in MATLAB 8.1 (R2013a)). This resulted in orthogonalized functions (e.g., for condition 1: *X'*_*E1‐Theta‐Anterior*_ and *X'*_*E1‐Theta‐Posterior*_). We conducted two separate EEG‐informed fMRI analyses (one each for anterior and posterior theta activity, GLM analyses performed on the 3‐dimensional functional volume time courses, see above) using two different design matrices, each of which contained three (one per condition) of the modeled BOLD response functions corresponding to the orthogonalized regressors (e.g., for anterior theta: *X'*_*E1‐Theta‐Anterior*_, *X'*_*E2‐Theta‐Anterior*_, and *X'*_*E3‐Theta‐Anterior*_) and all of which contained modeled BOLD response functions corresponding to *X*_*E1*_, *X*_*E2*_, and *X*_*E3*_, the unchanged regressors for the early and late maintenance and the retrieval period (as defined in the section *fMRI parameter and analysis*) and six translation and rotation vectors derived from the motion correction (confounds).

Two different event‐related analyses were then performed for each subject (one for each of the ROIs) revealing the beta estimates of each regressor of the GLM. The resulting statistical maps were entered into a second‐level group analysis treating intersubject variability as a random effect. For each analysis the contrast images containing all beta values of the regressors representing theta activity evoked by encoding stimuli (e.g., for anterior theta: *X'*_*E1‐Theta‐Anterior*_, *X'*_*E2‐Theta‐Anterior*_, and *X'*_*E3‐Theta‐Anterior*_) were calculated. We set a cluster‐defining threshold (CDT) of *p* < 0.001 and applied the “Cluster‐level Statistical Threshold Estimator” plugin included in BrainVoyager which uses Monte Carlo simulations for estimating the minimum cluster size for inferences. For all EEG‐informed GLMs this resulted in a minimum cluster size of 22 voxels using an alpha of 0.05 and 1000 iterations. Only regions exceeding this threshold were reported as significant activations.

Additionally, single‐factor repeated measures ANOVAs were calculated, including the beta estimates of the regressors representing theta‐specific activity during the encoding period. All resulting clusters were defined as Volumes‐of‐Interest (VOI) and mean beta estimates were extracted per subject and condition. The mean estimates of the clusters were compared between each condition using *t*‐tests.

### 2.8 Data Quality Assessment

To be sure that the measured theta power signals were not crucially superimposed by movement artifacts as it was shown in previous work (Fellner et al. 2016), we performed several checks of the relation between the subjects' movements and the theta power signals. For this purpose, we first normalized the 6 motion regressors (change in motion relative to the first volume), then calculated the amount of the difference between the measure of one volume and the preceding one (change in motion from volume to volume). The sum of movement changes in the 6 motion regressors finally resulted in a motion measure *mp* (adopted from Fellner et al.) calculated for every subject. To evaluate motion occurring around the encoding phase, we used the sum of the values of *mp* of the volume containing the last stimulus of an encoding phase and the following volume.

First, it was checked whether there were differences in movement during the encoding phases between the three WM loading conditions. Repeated measures ANOVA comparing averaged motion measures during the encoding phase from all three WM load conditions indicated no differences between the conditions (*p* = 0.76).

Furthermore, the relationship between motion and theta power values from anterior (i) and posterior (ii) electrodes were analyzed in two separate linear‐mixed effect models (LMM). The models included the motion measures surrounding the encoding phase (*mp*_*enc*_) and the WM load condition as fixed factors, the interaction between these two and subject as a random intercept to account for repeated measures. Evoked theta power values were entered as dependent variables. Neither the motion factor *mp*_*enc*_ (*p*_*anterior*_ = 0.94, *p*_*posterior*_ = 0.29) nor the interaction *mp*_*enc*_ × WM load (*p*_*anterior*_ = 0.75, *p*_*posterior*_ = 0.30) showed significant effects on the theta power values, indicating no substantial influence of the motion on the measured values.

### 2.9 Statistical Analyses

All statistical analyses were performed using the SPSS software package (26.0). Repeated measures ANOVAs were calculated for reaction times (correct answer within a timeframe of 6 s after presentation of the retrieval stimulus) and numbers of successfully encoded items in order to check for significant WM load effects. For theta power peaks and latencies two‐factor repeated measures ANOVAs (ROI × WM load) were used. In case of a significant Mauchly‐test, we used the Greenhouse–Geisser correction. Post hoc tests for differences between conditions were done using *t*‐tests or Wilcoxon‐tests, if data was not normally distributed. In order to check for relationships between behavioral and biological measures, we performed correlational analyses for each WM load condition between the number of successfully encoded items and mean beta estimates for EEG‐fMRI VOIs as well as mean theta peak power values using Spearman's rank correlation coefficients including a Bonferroni correction for multiple testing.

## 3 Results

### 3.1 Behavioral Results

**FIGURE 2:**
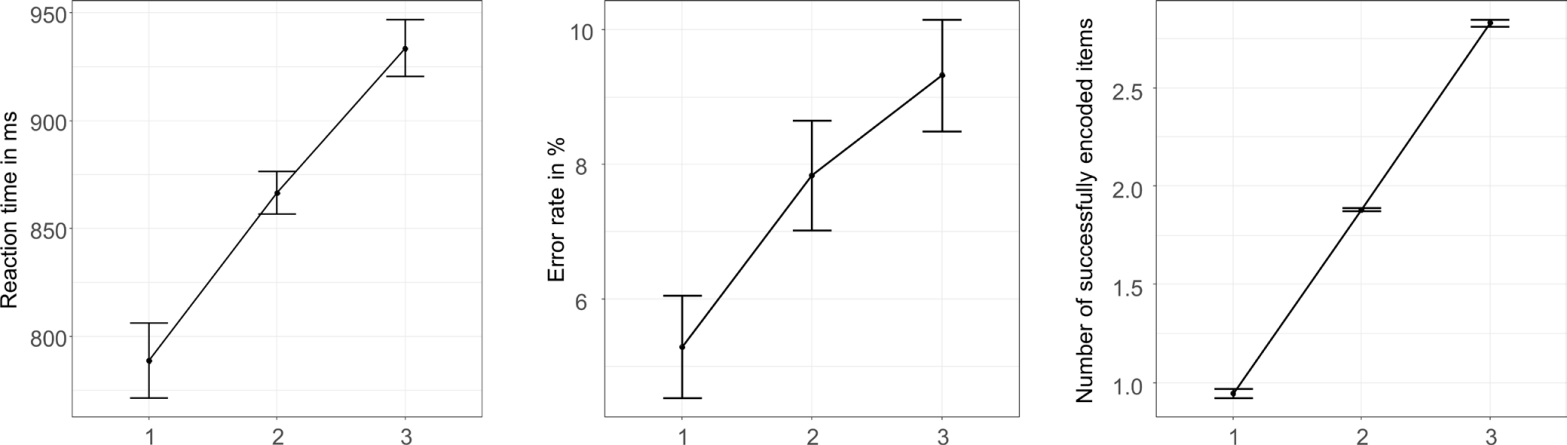
Behavioral results. Error bars represent standard errors of the mean.

As shown in Figure 2 there were significant main effects of the WM load condition on reaction times (*F*[1.4, 42.8] = 27.0, *p* < 0.001, Greenhouse–Geisser [GG] corrected), error rates (*F*[2, 60] = 6.4, *p* = 0.003) and number of successfully encoded items (*F*[1.1, 32.1] = 2842.7, *p* < 0.001, GG corrected). Post hoc Wilcoxon tests revealed significantly increased reaction times (*Z* = −3.6, *p* < 0.001), error rates (*Z* = −2.1, *p* = 0.038) and numbers of successfully encoded items (*Z* = −4.9, *p* < 0.001) for condition 2 in comparison with condition 1, significantly increased reaction times (*Z* = −4.3, *p* < 0.001) and numbers of successfully encoded items (*Z* = −4.9, *p* < 0.001) for condition 3 in comparison with condition 2, and significantly increased reaction times (*Z* = −4.3, *p* < 0.001), error rates (*Z* = −3.1, *p* = 0.002) and numbers of successfully encoded items (*T* = −4.9, *p* < 0.001) for condition 3 in comparison with condition 1.

### 3.2 EEG Results

**FIGURE 3:**
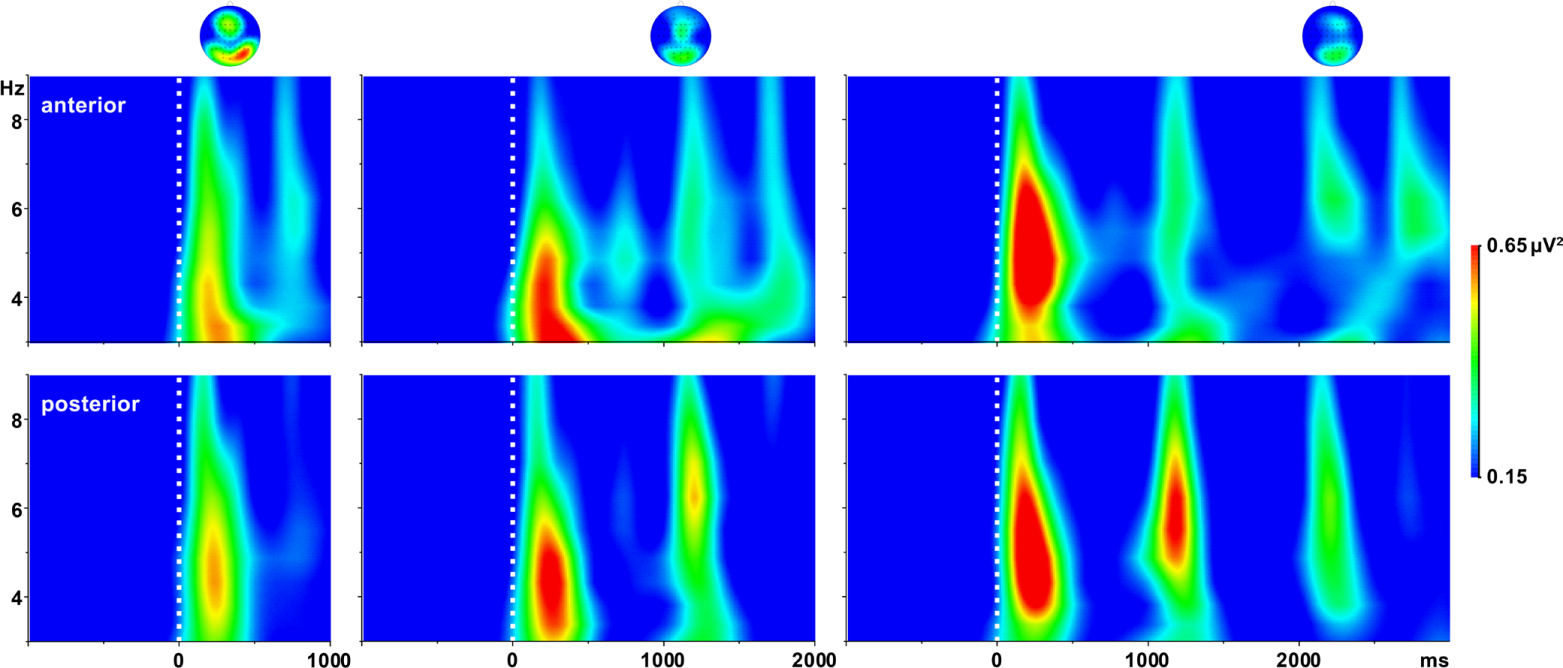
Grand‐averages of evoked theta responses to visual encoding stimuli measured at anterior (top row) and posterior (bottom row) pools of electrodes. The upper part shows the scalp topographies at 5 Hz between 200 and 250 ms following the first, second, and third stimuli, respectively.

**FIGURE 4:**
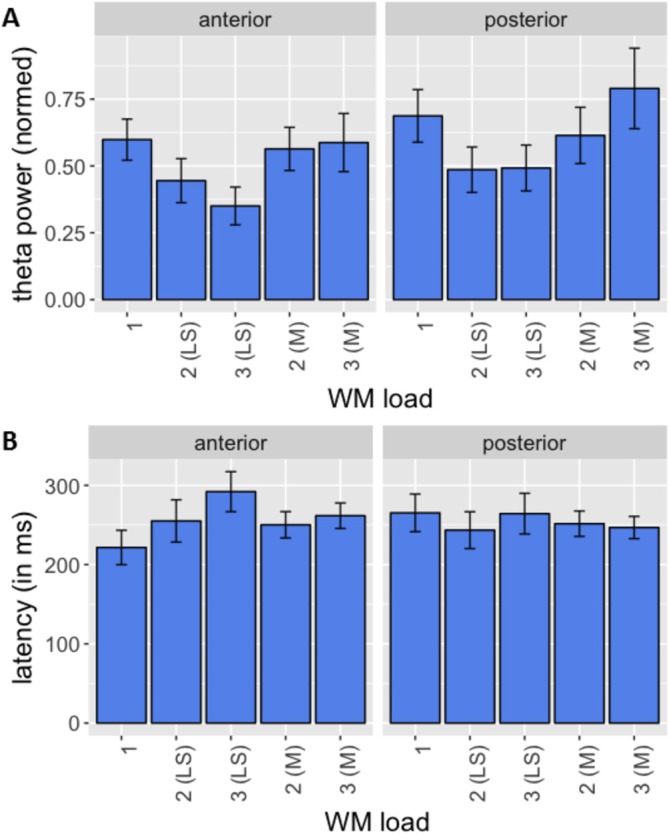
Group mean theta power peak amplitudes (A) and latencies (B) measured at anterior and posterior pools of EEG electrodes. For conditions 2 and 3 values following the last stimulus (LS) and means (M) of the values following all stimuli are shown. Error bars represent standard errors of the mean.

The presentation of the visual encoding stimuli elicited electrophysiological responses within the theta‐frequency range. With respect to theta power peaks evoked by the last encoding stimulus of each condition, there was no significant interaction between electrode ROI site and WM load, and no significant main effect of electrode ROI site. However, we found a significant main effect of WM load (*F*[2, 44.8] = 6.4; *p* = 0.007, GG corrected). Post hoc Wilcoxon tests revealed significantly lower theta values for condition 2 compared to condition 1 for anterior (*Z* = −2.0, *p* = 0.046) and posterior electrodes (*Z* = −1.8, *p* = 0.002). Moreover, theta values for condition 3 were significantly lower compared to condition 1 for anterior electrodes (*Z* = −3.1, *p* < 0.001). With regard to theta peak latencies, there was no significant main effect of electrode site or WM load. This was also true for mean theta peak power values across all encoding stimuli presented in the respective condition. Theta power peaked between around 100 and 450 ms after stimulus presentation, with topographical maxima over anterior (fronto‐central) and posterior (parieto‐occipital) electrodes (see Figure 3). Power amplitudes and latencies are depicted in Figure 4. Since the focus of this paper was on the analysis of theta‐oscillations associated networks during the encoding phase, we omitted analysis and reporting of oscillatory activity during the maintenance and retrieval phase.

### 3.3 Conventional and EEG‐Informed fMRI Results

**FIGURE 5:**
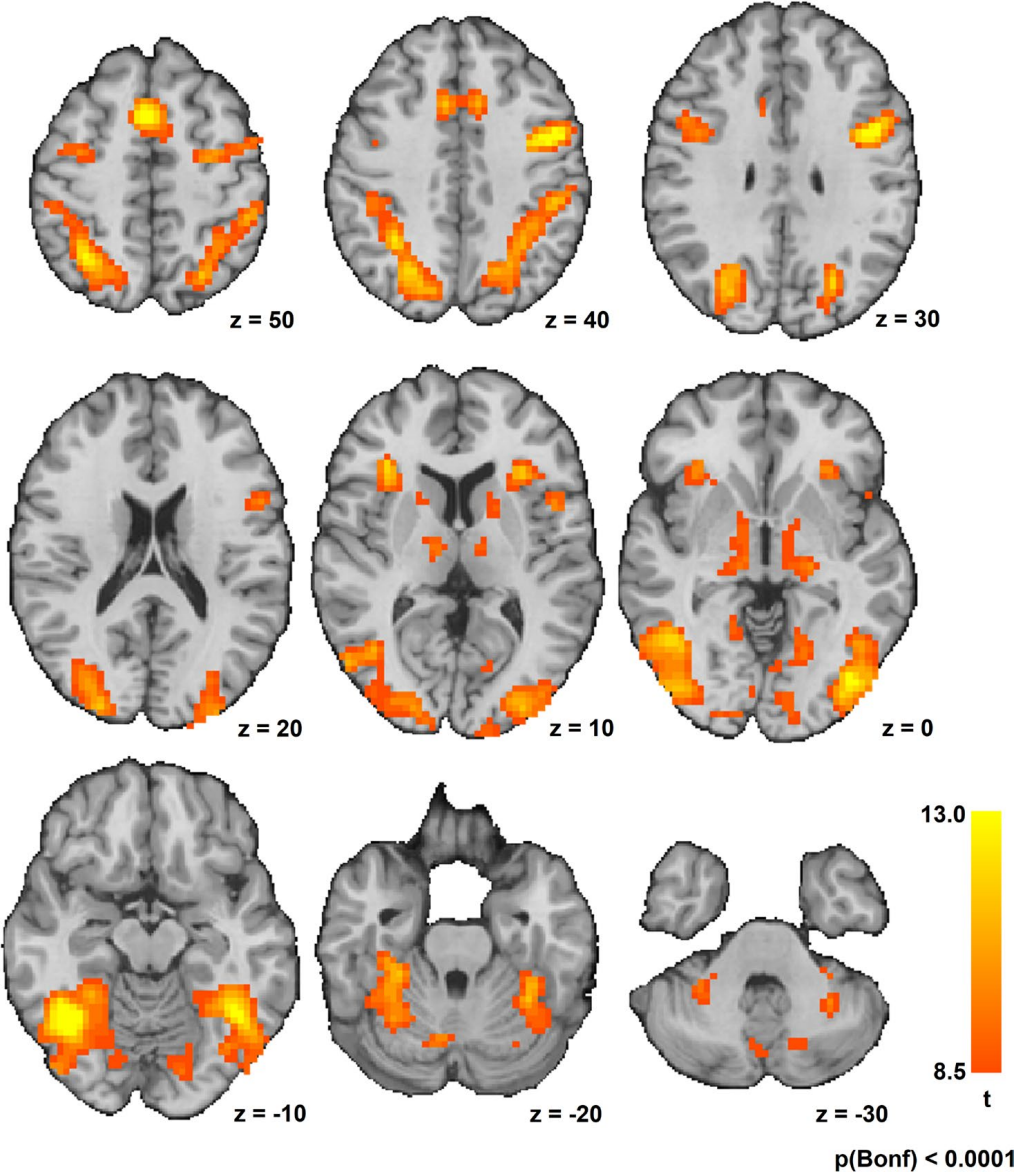
Conventional fMRI Analysis: BOLD response to encoding stimuli. Random effects analysis, *p* < 0.0001 (Bonferroni corrected for multiple comparisons). Images are displayed in radiological convention at Talairach coordinates as specified in the figure.

The conventional fMRI analysis mainly revealed fronto‐parietal regions, bilateral insulae, bilateral occipital regions, and the thalamus to be active during visual working memory encoding. Detailed results are depicted in Figure 5. A WM load dependent change was also found in some fronto‐parietal regions and occipital areas (see Table 1). *T*‐Tests revealed that the differences of mean beta estimates of the clusters were driven by a significant increase of beta estimates from condition 1 to 2 and significant increases of beta estimates between condition 1 and 3. The mean beta estimates of all clusters were higher in condition 3 than in condition 2, but these differences were not statistically significant.

**TABLE 1:** Conventional fMRI analysis: Brain regions with significant WM load dependent change of fMRI activity during visual WM encoding (random effects analysis, *p* < 0.0001, Bonferroni corrected for multiple comparisons).

Localization peak voxel	Talairach coordinates			*T*_max_	*T*_mean_	Number of voxels	Brodmann area	Regions included
	*x*	*y*	*z*					
Conventional analysis								
Left precentral gyrus	−44	1	29	87.40	57.79	44	6	IFG, MFG
Right superior parietal lobule	24	−60	46	49.47	48.14	6	7	Precuneus
Left inferior parietal lobule	−31	−45	44	47.69	46.85	3	40	—
Right fusiform gyrus	32	−44	−13	96.22	56.40	387	37	Cb, IOG, ITG, LiG, MOG, MTG, PHG, PCu
Left fusiform gyrus	−37	−71	−9	86.05	56.29	421	19	Cb, IOG, ITG, LiG, MOG, MTG, PCu, SOG

Abbreviations: Cb, cerebellum; IFG, inferior frontal gyrus; ITG, inferior temporal gyrus; LiG, lingual gyrus; MFG, medial frontal gyrus; MOG, middle occipital gyrus; MTG, middle temporal gyrus; PCu, precuneus; PHG, parahippocampal gyrus; SOG, superior occipital gyrus.

**FIGURE 6:**
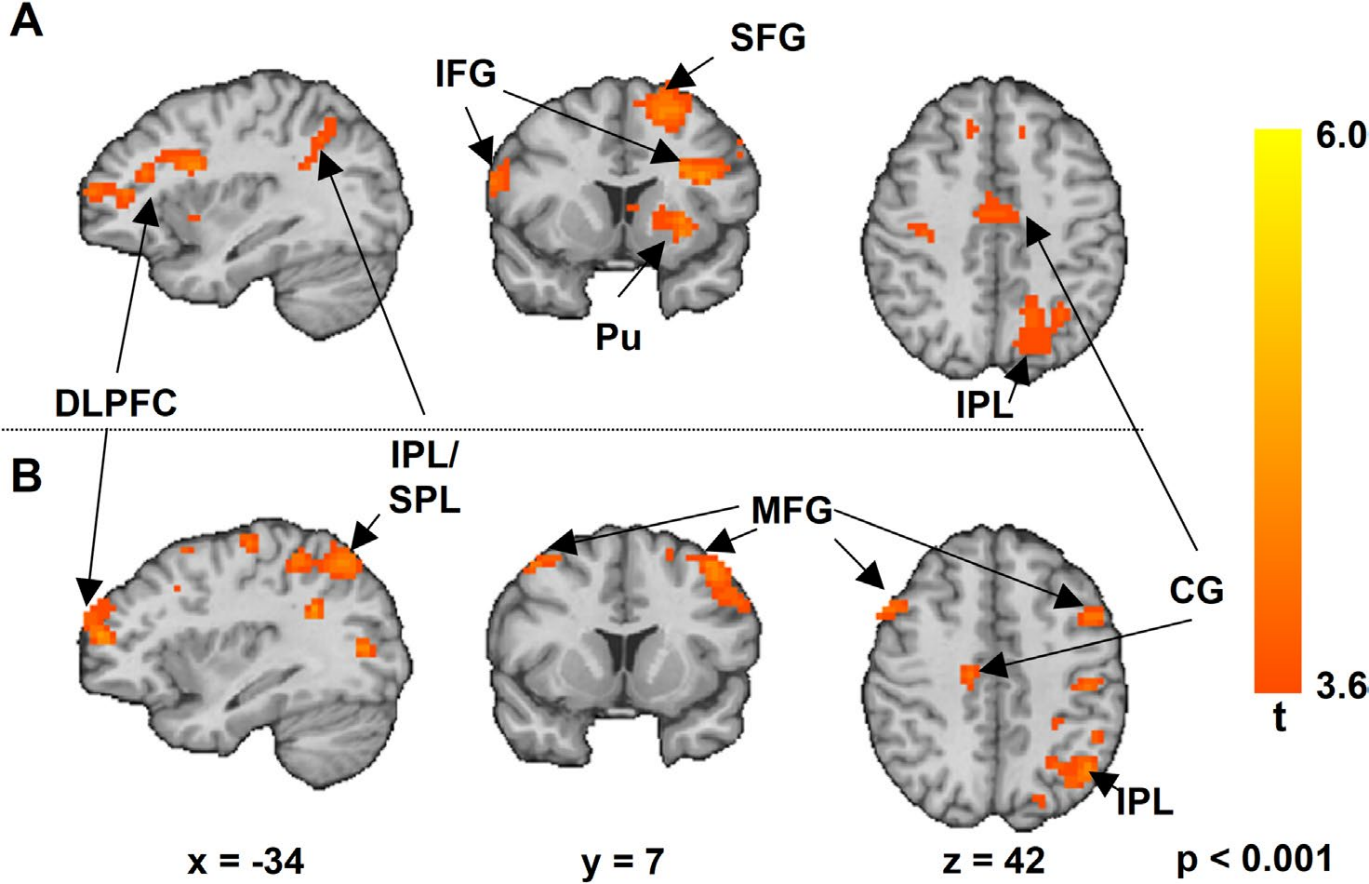
EEG‐informed fMRI Analysis: *Frequency‐*specific BOLD response to stimuli during encoding revealed by single trial coupling of *Theta* power variation from anterior (A) and posterior (B) electrodes. Random effects analysis, *p* < 0.0001 (uncorrected for multiple comparisons). Images are displayed in radiological convention at Talairach coordinates as specified in the figure. CG, cingulate gyrus; DLPFC, dorsolateral prefrontal cortex; IFG, inferior frontal gyrus; IPL, inferior parietal lobule; MFG, middle frontal gyrus; Pu, Putamen; SFG, superior frontal gyrus; SPL, superior parietal lobule.

The EEG‐informed fMRI analysis revealed a fronto‐parietal working memory encoding network mediated by theta oscillatory activity. Frontal brain areas involved in the network comprised regions within caudal and rostral parts of the bilateral dorsolateral prefrontal cortex (DLPFC), the bilateral inferior frontal gyrus, the left dorsal premotor cortex, the supplementary motor area, and the left precentral gyrus. In addition, we found theta‐related activity during encoding within left superior and inferior parietal regions, the left middle temporal gyrus, and the right occipital cortex. Theta activity measured over anterior EEG electrodes was additionally associated with activity within the right precentral cortex, the posterior cingulate cortex, and the left putamen, whereas theta activity measured in posterior electrodes was associated with activations in left and right parietal regions. Detailed results are depicted in Figure 6 and Table 2.

**TABLE 2:** EEG‐informed fMRI analysis: Brain regions with significant increase of theta‐specific fMRI activity during visual WM encoding (random effects analysis, *p* < 0.0001, uncorrected for multiple comparisons).

Localization peak voxel	Talairach coordinates			*T*_max_	*T*_mean_	Number of voxels	Brodmann area	Regions included
	*x*	*y*	*z*					
Anterior theta								
Right superior frontal gyrus	11	29	45	4.29	3.85	38	BA 8	
Left middle frontal gyrus	−35	37	14	5.18	4.09	64	BA 10	Left rostral LPFC
Left middle frontal gyrus	−35	30	23	4.94	4.10	24	BA 9	Left caudal LPFC
Right middle frontal gyrus	36	29	22	5.18	4.13	23	BA 46	Right caudal LPFC
Left inferior frontal gyrus	−35	9	24	5.81	4.17	177	BA 9	
Left posterior superior frontal gyrus	−22	12	54	5.40	4.22	191	BA 6	Left dorsal premotor cortex
Left medial frontal gyrus	−13	27	35	4.76	4.57	40	BA 6	
Left medial frontal gyrus	−11	−15	57	4.59	3.93	138	BA 6	Left posterior medial frontal cortex (SMA)
Right medial frontal gyrus	4	48	22	4.29	3.85	35	BA 9	
Left cingulate gyrus	−7	−9	41	4.82	4.00	57	BA 24	
Right insula	41	−27	18	4.27	3.86	27	BA 13	
Right precentral gyrus	64	−3	23	5.55	4.16	136	BA 6	Right IFG, right dorsal premotor cortex
Right precentral gyrus	32	−18	36	5.28	4.14	39	BA 4	
Right postcentral gyrus	61	−23	14	4.59	3.96	108	BA 40	
Left posterior cingulate gyrus	−20	−53	15	5.34	3.96	538	BA 30 (7, 40)	Left SPL, left IPL
Left posterior cingulate	−10	−35	24	4.55	3.92	86	BA 23	
Right superior temporal gyrus	50	−9	2	4.43	3.92	27	BA 22	
Left middle temporal gyrus	−55	−45	−4	4.66	4.10	25	BA 37	
Right fusiform gyrus	49	−42	−9	4.64	3.98	34	BA 37	
Right thalamus	8	−1	8	4.03	3.82	34	—	
Right cuneus	16	−78	18	4.83	4.05	38	BA 18	
Left putamen	−28	8	3	5.39	4.11	151	—	
Posterior theta								
Left superior frontal gyrus	−35	49	17	5.13	4.05	155	BA 10	Left rostral LPFC
Right middle frontal gyrus	40	6	52	4.78	4.06	109	BA 6	Right caudal LPFC
Right middle frontal gyrus	37	37	33	4.68	4.02	58	BA 9	
Left insula	−44	−18	−1	4.21	3.88	22	BA 13	
Left precentral gyrus	−56	−3	29	5.00	4.02	431	BA 6	
Right postcentral gyrus	23	−32	64	5.78	4.08	646	BA 3	Right precentral gyrus (BA 4)
Right supramarginal gyrus	61	−41	29	4.15	3.83	55	BA40	
Left inferior parietal lobule	−41	−60	47	4.88	3.99	246	BA7 (40)	
Right postcentral gyrus	62	−21	17	4.98	3.93	136	BA 40	Right inferior parietal lobule
Left precuneus	−25	−76	33	4.78	4.01	104	BA 19	
Right precuneus	32	−72	20	4.40	3.88	28	BA31	
Left middle occipital gyrus	−34	−72	11	4.68	3.94	35	BA19	

Abbreviations: IPL, inferior parietal gyrus; LPFC, lateral prefrontal cortex; SPL, superior parietal gyrus.

Load dependent changes of theta‐specific fMRI activity could only be discovered using activity measured in posterior electrodes (see Table 3). These changes were found in the left DLPFC, left insula, right precentral, and bilateral occipital areas. The mean beta values of condition 3 compared to condition 1 and also compared to condition 2 were higher in all clusters. Additionally, the beta values of condition 2 compared to condition 1 were higher in a cluster including the right precentral cortex and some parietal areas.

**TABLE 3:** EEG‐informed fMRI analysis: Brain regions with significant WM load‐dependent change of theta‐specific fMRI activity during visual WM encoding (random effects analysis, *p* < 0.0001, uncorrected for multiple comparisons).

Localization peak voxel	Talairach coordinates			*F*_max_	*F*_mean_	Number of voxels	Brodmann area	Regions included
	*x*	*y*	*z*					
Posterior theta								
Right medial frontal gyrus	7	−3	50	10.62	8.95	23	BA 6	
Left insula	−40	−12	9	13.89	10.04	27	BA 13	—
Left insula	−25	−18	20	12.30	8.67	33	BA 13	
Left precentral gyrus	−47	−11	28	10.44	8.80	29	BA 6	
Right precentral gyrus	14	−30	66	17.47	9.77	447	BA 4	MFG, paracentral lobule, postcentral gyrus, IPL, SPL
Left postcentral gyrus	−44	−20	42	10.24	8.55	25	BA 3	
Left inferior parietal lobule	−41	−38	55	11.34	8.95	33	BA 40	
Left superior temporal gyrus	−52	−54	5	11.39	9.02	57	BA 39	—
Left middle occipital gyrus	−35	−83	4	15.01	10.18	50	BA 19	—
Right lingual gyrus	16	−78	2	21.00	10.11	591	BA 18	Cuneus

Abbreviations: IPL, inferior parietal gyrus; MFG, medial frontal gyrus; SPL, superior parietal gyrus.

There was no significant correlation between beta estimates extracted from VOIs or EEG theta measures with WM performance.

## 4 Discussion

The present study simultaneously recorded EEG‐fMRI data in order to investigate the brain areas associated with theta oscillatory activity during WM encoding in a delayed matched to sample WM task. Thus, for the first time, a brain network specifically related to activity in the theta‐frequency range in the encoding of visual information into WM has been described.

A bilateral fronto‐parietal network is known to be associated with WM processes according to an extensive body of evidence from fMRI studies (Daniel et al. 2016; Li et al. 2022; Rottschy et al. 2012). Our results confirm the involvement of theta oscillatory activity in this network. Most of the brain regions associated with the theta‐specific WM encoding have consistently been shown to be part of this WM network: the rostral and caudal LPFC, the inferior frontal gyrus, the SMA, the dorsal premotor cortex, the parietal cortex, the fusiform gyrus, and the dorsal striatum. The inferior frontal gyrus and the parietal regions found in our study have also been shown to be specifically involved in visual WM encoding (Bittner et al. 2015). In line with meta‐analytical observations on specific components of WM tasks, the theta‐specific network revealed in our study comprised regions within the SMA and the lateral dorsal premotor cortex (superior frontal gyrus). These brain regions have been reported to be active in WM studies using non‐verbal stimulus material (e.g., objects or shapes like in our experiment) rather than in studies using verbal stimulus material. Moreover, our theta‐informed fMRI analysis revealed robust activations of the bilateral inferior frontal gyrus, which has been found to be recruited during memorizing of object identity (like in our study) but not object localization (Rottschy et al. 2012).

Previous EEG‐fMRI WM studies have also reported theta oscillatory activity to be related to WM networks. However, most of these studies used WM paradigms that did not allow the temporal separation of the different WM processes (encoding, maintenance, and retrieval) according to the conceptualization of Baddeley (2012) and its putatively distinct neural correlates (Forsyth et al. 2021; Qin et al. 2023; Sammer et al. 2007; Zhao et al. 2017), or investigated processes of WM maintenance or retrieval (Baenninger et al. 2016; Gomes et al. 2023; Herweg et al. 2016; Kottlow et al. 2015; Michels et al. 2010; Michels et al. 2012; Mizuhara et al. 2015; Scheeringa et al. 2009; Xu et al. 2024). Meltzer et al. did not find a correlation between frontal midline theta activity and fMRI BOLD signal during the encoding of digits in a Sternberg task, but have not recorded EEG and fMRI simultaneously (Meltzer et al. 2007). Using a joint independent component analysis of simultaneously recorded EEG‐fMRI n‐back WM data, Zhao et al. found a WM load‐dependent theta response evoked in the early stage after stimulus onset corresponding to an enhanced activation in frontal and parietal brain regions (Zhao et al. 2017). Other simultaneous EEG‐fMRI studies revealed a positive correlation of frontal midline theta with the right superior frontal gyrus (Forsyth et al. 2021; Qin et al. 2023) or SMA (Qin et al. 2023) and load‐dependent theta power changes in prefrontal regions. However, as the n‐back task is not able to temporally distinguish between different WM processes, it remains unclear whether the theta‐related brain activity reported in these studies is specifically related to WM encoding. We have identified very similar regions within the DLPFC (Forsyth et al. 2021; Li et al. 2019; Zhao et al. 2017), the SMA (Qin et al. 2023) and the parietal cortex (Zhao et al. 2017) as part of the theta‐specific network found in our study, while clearly investigating theta‐related encoding processes. This suggests that synchronization of theta oscillatory activity between these regions, which have been shown to be active during the maintenance of memory, too (Linden et al. 2003), is already involved during the encoding of information. This is in line with the assumption that encoding is a rate‐limiting process underlying our memory capacity limit (Muthukrishnan et al. 2020), which is underlined by reports from an EEG study that oscillatory theta activity only during encoding but not during maintenance or retrieval periods predicted the number of successfully memorized items (Haenschel et al. 2009). Accordingly, our results suggest that the formation of a core WM network known from meta‐analytical evidence including IFG, posterior dorsal and medial frontal cortex, and lateral parietal lobe (Owen et al. 2005; Rottschy et al. 2012; Wager and Smith 2003) might take place during the encoding process facilitating theta synchrony as a binding mechanism. This matches the assumption that encoding of sensory information is different from bottom‐up perceptual processing rather representing the ability to convert sensory stimuli into a maintainable construct when the stimulus is no longer accessible (Palva et al. 2011; Woodman and Vogel 2005).

The presentation of the visual encoding stimuli elicited electrophysiological responses within the theta‐frequency range with topographical maxima over anterior (fronto‐central) and posterior (parieto‐occipital) electrodes in line with previous findings (Haenschel et al. 2009; Koychev et al. 2011). Thus, despite the difficulties of recording oscillatory activity in an fMRI environment, we were able to the inform our fMRI analysis with time courses of theta activity mirroring previous findings from EEG studies. However, despite the finding of one cluster centered within the right postcentral gyrus and two small clusters within the left precuneus in the EEG‐fMRI analysis using theta time courses derived from posterior electrodes, we found similar regions in both analyses. As fMRI analyses informed by EEG oscillatory activity offer insights into connectivity mechanisms of brain networks (Leicht et al. 2021), this finding strengthens the assumption that both analyses target one single network facilitating synchronization of theta oscillatory activity to be connected, which projects theta activity to two clusters of frontal‐central and parieto‐occipital electrodes, respectively.

The importance of oscillatory theta activity for WM encoding is supported by a large body of evidence (e.g., Backus et al. 2016; Dai et al. 2017; Haenschel et al. 2009; Kottlow et al. 2015), although other frequencies seem to be involved, too (Palva et al. 2011). A recent study showed that out of seven different EEG frequency bands only the theta band varied significantly in 13 brain connections due to memory load during visual WM encoding (Muthukrishnan et al. 2020). In this study, changes in theta coherence with other brain regions due to an increase in visual WM load were observed in areas such as the inferior parietal lobule (BA 40), the fusiform gyrus (BA 37) and the lingual gyrus (BA 18), which are also part of the theta‐specific network revealed in our study. The IFG, which revealed the highest *t*‐values amongst the regions involved in the theta‐specific WM encoding network detected in our EEG‐informed fMRI analysis, has also been reported as a cortical source of theta activity during visual WM encoding by means of EEG source localization (Jaiswal et al. 2010). Theta oscillations have already been discussed to coordinate different brain regions during working memory processes by long‐range synchronization (Sauseng et al. 2010) and interregional phase synchronization (Fuentemilla et al. 2014). A study using intracranial EEG recordings found a load‐dependent involvement of theta oscillatory activity in WM processes in similar prefrontal regions as identified in our study (Brzezicka et al. 2019). Against this background, the results of our study provide further evidence for the concept that functional binding of widely distributed cortical assemblies in the theta frequency range might represent a crucial mechanism for WM.

Some limitations of the current study need to be taken into account when considering its findings. According to our hypothesis based on strong evidence on a crucial role of theta oscillations in WM, we did not investigate frequencies other than theta oscillations, although there might have been effects during encoding in higher frequencies such as the beta or gamma frequency range (Haenschel et al. 2009). However, the investigation of high frequencies in simultaneous EEG‐fMRI data faces more difficulties. This is particularly true for the current study as we decided to use an EEG amplitude resolution of 0.5 μV during recording in order to avoid clipping in EEG data during MR gradient artifacts, which would have precluded a proper gradient artifact and subsequently cardioballistic artifact cleaning. In contrary to our hypothesis, the activity of the theta‐specific brain network was not related to WM performance in our study. This might be due to the high number of subjects (48% in condition 1 and 39% in conditions 2 and 3) with maximum numbers of successfully encoded items (ceiling effect) reducing the variance in our behavioral data. Thus, in future studies, the implementation of a condition involving a higher WM load has to be considered. Another limitation, which applies to EEG‐fMRI studies in general, could be that EEG activity is influenced by movement of the subjects. Even small movements can cause changes in the EEG signal, which could later be mistaken as neuronal activity (Fellner et al. 2016). However, since our EEG results are largely consistent with data measured outside the scanner (Haenschel et al. 2009) and we could not find any statistical correlation between the amount of movement and the measured theta power, this issue does not affect our results crucially.

WM deficits occurring in neuropsychiatric disorders such as schizophrenia seem particularly to involve the encoding of information (Haenschel et al. 2007; Hahn et al. 2010; Mayer et al. 2014; Stablein et al. 2019). Findings from fMRI studies suggest that impaired encoding of visual content is associated with reduced neuronal activation in lateral prefrontal and parietal brain regions also found as part of the theta‐specific network revealed in our study (inferior and middle frontal gyrus, lateral parietal cortex) (Anticevic et al. 2013; Bittner et al. 2015; Karch et al. 2009). Electrophysiological studies revealed a crucial pathophysiological contribution of alterations of theta oscillatory activity during WM encoding in patients with schizophrenia (Haenschel et al. 2009). Thus, recent findings showing the potential of transcranial alternating current stimulation (tACS) in enhancing WM performance might present a new treatment option for schizophrenia and other neuropsychiatric diseases (Elyamany et al. 2021; Rauh et al. 2023; Reinhart and Nguyen 2019). The results of our study could contribute to the development of an individualized tACS approach by offering the possibility to identify individual peak frequencies within the theta frequency range and individual tACS target regions by means of simultaneous EEG‐fMRI. While we used larger clusters at a group level to identify regions associated with theta oscillations during the encoding process, this approach could be adapted for the purpose of individual targeting. Theta information could be extracted from individual clusters in the EEG and fed into a single‐subject fMRI analysis to identify target regions for the stimulation. Subsequently, algorithms for the optimized back projection from the target regions onto the electrode level (Rauh et al. 2023) could be applied for an optimized individual stimulation approach.

## 5 Conclusion

Using simultaneously recorded EEG and fMRI, this study revealed a brain network specifically related to cortical oscillatory activity in the theta‐frequency range observed during the encoding of visual information into WM. Our results give reason to assume that the formation of the WM network extensively described in fMRI studies including IFG, posterior dorsal and medial frontal cortex, and lateral parietal lobe might take place during the encoding process utilizing theta synchrony as a binding mechanism.

## Conflicts of Interest

The authors declare no conflicts of interest.

## Data Availability

The data that support the findings of this study are available from the corresponding author upon reasonable request.
